# Building Materials Made of Wood Waste a Solution to Achieve the Sustainable Development Goals

**DOI:** 10.3390/ma14247638

**Published:** 2021-12-11

**Authors:** Dorin Maier

**Affiliations:** Faculty of Civil Engineering, Technical University of Cluj-Napoca, 400020 Cluj-Napoca, Romania; dorin.maier@ccm.utcluj.ro

**Keywords:** sustainable development goals, wood waste, construction waste, wood materials, building materials

## Abstract

In order to reduce the impact of human activities on the environment, in 2015, the United Nations launched the 2030 Agenda for Sustainable Development, proposing 17 Sustainable Development Goals with 169 associated targets. It is well-known that the construction industry is a major contributor to global CO_2_ emissions, and if a solution to reduce construction activity is not possible, considering the increasing population, then other solutions must be developed to decrease their negative environmental impact. In this context, the purpose of this paper is to investigate whether the use of wood waste as a building material can be a solution to achieve the Sustainable Development Goals. The research procedure included a bibliometric literature search, a scientometric analysis and an in-depth discussion. The analysis was done with the help of the software VOSviewer and Bibliometrix; the data were extracted mainly from the ISI Web of Science database. The extraction of data was done using the PRISMA method, and thus a sample of 212 peer-reviewed journal articles was established. The main results indicate an increasing interest in this topic in the last several years, as well as a switch from considering wood waste as just a source to generate heat and energy to the use of wood waste as a building material. The main uses of wood waste as a building material are in the composition of particleboards and in various mortar and concrete mixtures. The field of wood waste has many potential directions towards future development, and if the immense treasure represented by the forests, and implicitly the wood, is used efficiently, it can be a good solution to the problem of sustainable development of society.

## 1. Introduction

Modern society, in its rapid race for development and prosperity, is faced with new and more demanding challenges on all levels. Environmental protection and issues regarding climate change are some of the challenges that are becoming more and more present in our everyday life. The construction industry is one of the human activities that puts a lot of pressure on the environment, as it needs to deliver more and better spaces for a growing population. The process of construction also generates pollution and waste all over the world. In this context, there is a need to find new and better ways to construct while having a lower impact on the environment.

As a response to all of the new challenges of modern society, in 2015, the United Nations launched the 2030 Agenda for Sustainable Development [1] with the purpose of ending poverty and set the world on a path of peace, prosperity, and opportunity for all on a healthy planet. At the core of the 2030 Agenda are the 17 Sustainable Development Goals (SDGs) and 169 associated targets. The Sustainable Development Goals cover a wide range of societal concerns, such as ending poverty; ensuring gender equality and quality education for all; sanitation; climate action; providing water and maintaining healthy wellbeing; energy and environment; and peace and social justice. The European Commission report from 2020 [2] emphasizes the interconnection of the goals and highlights the importance of global partnership for their successful implementation. The latest reports [3] show that there are delays in the achievement program. In this context, the purpose of this research is to analyze and identify solutions to help the achievement of these goals from a construction industry point of view.

People are more aware of the higher urban living standards offered by cities, and consequently, there is an increased pressure on cities to develop and grow their size so as to receive more people. In a report by the United Nations [4], it is shown that of the total world population, half live in cities, and some of the projections indicate an increase of 60% in the urban population through 2030. The paradox is that cities occupy only 3% of the Earth’s surface, but they use 60% of resources, produce 75% of global carbon emissions and consume 60–80% of global energy. All of this has a serious environmental and social impact, and thus an urgent need arises to find green building materials that can help the achievement of sustainable development [5].

It is known that the construction industry is a major contributor to the global CO_2_ emissions, mainly through embodied and operational energy use, and thus there must be developed solutions to decrease the negative impact of its activity on the environment. If a solution of less construction is not possible due to the increasing population, then better building solutions must be developed. The construction process implies the use of large quantities of natural resources, and at the same time, some of the construction products have a negative effect on the indoor environment and human health [6]. Concrete is the most used building material due to its many advantages and ease of use. The other principal buildings materials are steel and ceramics blocks. All of them have a major disadvantage: they need high energy quantities in their production process. The only ecological building material with low energy consumption is wood. It not only needs low quantities of energy to be produced, but it also traps CO_2_ during the life of its structure.

Forests are a huge treasure as sought after and necessary as other sources of raw materials. Tree trunks and crowns are true accumulators of solar energy and stores of precious organic matter. Wood has been used since the earliest times of human existence, sharing with stone and clay the glory of birthing the first tools, the first homes and the first means of human defense. Wood is a natural, renewable building material, having an inhomogeneous (anisotropic) characteristic, made up of a large number of plant cells organized into specialized tissues, also called anatomical elements, which are very diverse. They differ in their lifetime and tree functions, shape and size, position in the tree and quantity or number. Many of the cells die during the life of the tree, retaining only the role of ensuring the mechanical strength of the tissues in its composition. The way in which the anatomical elements observable with the naked eye are grouped is called the macroscopic structure. It is important to know this because, depending on its appearance, the various species of wood can be identified and the most convenient areas of technological use of the wood can be established.

Before advancing with the discussion, it is important to highlight that we need to speak about using sustainable wood in construction, meaning that we need to know the origin of the wood elements. In the overall context of the research, the achievement of the Sustainable Development Goals, it is important not to solve a problem by using wood in the construction process and thereby create another problem, i.e., massive illegal deforestation. In this sense, the use of certified wood materials, wood that was harvested responsibly from well-managed forests that are continuously replenished, is mandatory, and we must ensure that there is no damage to the surrounding environment or to native flora and fauna.

A major challenge for the construction industry is also the management of waste produced in the building stages or in the demolition cases. To have an idea about the size of the phenomenon, in [7], it is stated that construction and demolition waste is the largest waste stream worldwide (30–40% of total solid waste). According to a report from Eurostat [8], in the European Union, the construction industry is the biggest contributor to waste, accounting for 36% of the total solid waste produced in 2018. In the case of the United States, this proportion was close to 67% (534 million tons) [9], and in China, the percent was 30–40% (2.36 billion tons) [10,11]. Given the negative impact of construction waste on the environment and the large amount of waste generated by building activities, finding solutions to better manage and use the waste is gathering more and more importance.

### Research Purpose

Considering the context described, the main research question of this study can be formulated as “*Are wood-based building materials, especially those made of wood waste, a solution to achieve sustainable development goals?*”.

In order to respond to this question, the research procedure included a bibliometric literature search, a scientometric analysis and an in-depth discussion. The data used for this study were extracted mainly from the ISI Web of Science database, while a small part was obtained from Scopus database. For a more coherent extraction of data, the PRISMA (Preferred Reporting Items for Systematic Reviews and Meta-Analyses) method was used. The literature highlights [12] the importance of the ability to analyze the evolution of scientific literature over time and also of revealing the intellectual relationships in the field.

The structure of the paper follows the structure of a scientific paper. After the short introduction and the purpose of the research, the methods and methodology of the study are presented. The next part of the paper is reserved for presentation of the main results obtained from conducting the research, and the responses to the research question are presented. The paper ends with the presentation of the main conclusions and with the list of bibliographic titles used in this study.

## 2. Materials and Methods

According to the research objectives, the research procedures include a bibliometric literature search, a scientometric analysis and an in-depth discussion. The bibliometric literature search is done in two parts. The first part is the process of interrogating the scientific database using certain keywords, and the second part is the process of screening and removing all irrelevant papers. The working plan of these methods includes the selection of articles in a sample database followed by filtering and refining the bibliographic data.

### 2.1. Data Collection

The main data used in this research were identified by using the ISI Web of Science database (ISI WoS). The interrogation was made using the keyword “wood waste”. The number of articles in the resulting search process are presented in Table 1.

After the preliminary keyword searching, it was necessary to screen the articles in order to select the articles most relevant to the purpose of the study. This process was done using the guidelines of the PRISMA method (Preferred Reporting Items for Systematic Reviews and Meta-Analyses) proposed by the researchers Moher et al. [13]. According to their research, the PRISMA approach indicates four steps to identify and extract the data: identification, screening, eligibility and inclusion.

In the first step of the PRISMA approach, the ISI WoS database was interrogated for articles having “wood waste” as their topic of research. The initial literature search was conducted in the beginning of October 2021. The search indicated a large number of articles, and by applying the filters in the PRISMA approach, 542 articles were processed.

In the screening step, from the initial number of articles were eliminated documents without author names and documents identified as book chapters, as well as documents that had a subject area other than environmental science, ecology, engineering, other science technology topics, construction building technology and other related topics. On this step, 47 articles were eliminated, resulting in a database of 495 articles.

The third phase of the PRISMA flow is checking the eligibility of the papers. Although this is the most time-consuming phase of the flow, the success of the research is directly influenced by this step. In this phase, the 495 papers were analyzed by reading the titles and abstracts to evaluate whether they corresponded to the purpose of this paper. The duplicates were eliminated first, mainly due to the return from both databases, followed by all the papers that did not correspond to the purpose of this study. The final number of articles included in the study was 212.

A scientometric analysis was conducted based on the selected 212 papers. The purpose of this analysis was to comprehensively understand the research field and to provide deeper insights than the ones previous studied.

### 2.2. Data Extraction and Analysis

In order to perform the analysis, the final database, formed by 212 journal articles, needed to be exported in a working format. The format of the export data file was chosen based on the files supported by the used software. In this research, for data analysis, two software were used, VOSviewer (version 1.6.17, developed by Nees Jan van Eck and Ludo Waltman at Leiden University’s Centre for Science and Technology Studies, Leiden, The Netherlands) and Bibliometrix (version 3.1, developed by Massimo Aria and Corrado Cuccurullo, Department of Economics and Statistics, University of Naples Federico II, Naples, Italy). The choice of these two software was made considering that both programs are freely available to the bibliometric research community. One of the main functionalities of the VOSviewer software is the possibility of displaying bibliometric maps, thus making it quite easy to interpret the data [14]. The information obtained by exporting the data from the scientific database contains the full range of resources available, including the title of the article, the author keywords, the keywords plus, the author’s name and the citation information, including the reference list of all the articles. In order to use the data in the software, the exported data need to be further analyzed and processed; this was done manually. The reason behind this is the fact that in order to generate accurate results, the VOSviewer and Bibliometrix software need a certain uniformity in the format information. This is also one of the reasons for working more with the ISI Web of Science database due to its greater uniformity in the exported data. The process of data standardization occupied a large percentage of the time needed to complete this study.

## 3. Results and Discussion

### 3.1. Number of Publications

**Figure 1:**
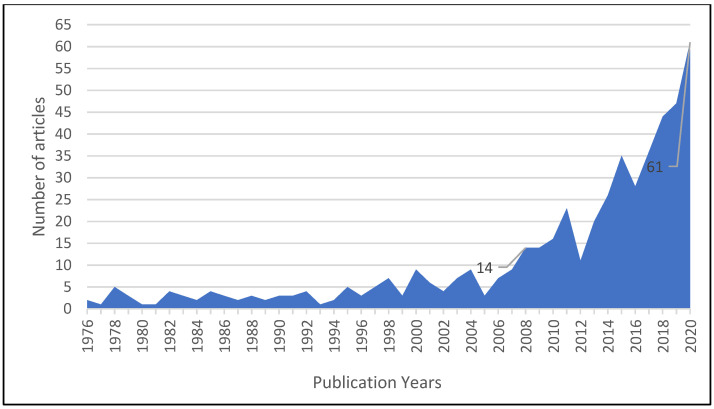
Annual evolution of the number of published articles on “wood waste” topic.

In the initial stage of research, the annual evolution of the published articles was analyzed (Figure 1). The purpose of this analysis is to identify the trend evolution of the researchers’ interest for subjects related to the use of wood waste. The graph from the Figure 1 was constructed using Microsoft Excel software, considering all the papers indexed in the ISI Web of Science, from 1975 to 2020. The grouping of the articles according to their publication years was taken directly from the ISI WoS database. The graph indicates on the *x*-axis the years of publication and on the *y*-axis the number of articles published each year.

The data from Figure 1 show that the number of articles addressing the topic of “wood waste” varies each year, but a general increasing trend can be seen. The annual evolution of the number of articles reveals that the number of articles published each year from 1976 to 2017 was under 10 articles per year. In 2008, the interest to this topic started to rise and 14 articles were published, and the trend continue to increase, reaching an amount of 61 articles published in 2020. The increasing trend of the number of articles can be explained by the increasing interest given to the protection of the environment in the last several years, and in this context, we can expect that the trend will continue to grow.

### 3.2. Journal Analysis

The interest for the subject of wood waste increased substantially in the last several years, especially due to the current situation characterized by a greater and greater awareness of the impact of human activity on the environment. The researchers focused their attention on these subjects and published their work in various journals. The journals with the largest numbers of articles on the wood waste subject at the moment of performing this search are presented in Table 2.

Data presented in Table 2 revealed that the journal with the highest number of published articles related to wood waste (36 articles) is the *Journal of Cleaner Production.* The second journal, in which a number of 31 articles were published, is *Waste Management*, and the third is the journal *Bioresources* with 22 articles. From the impact point of view, calculated mainly by the total citations obtained by each journal, the source with the greatest impact, with a total of 899 total citations, is the *Waste Management* journal. The second greatest journal impact is the *Journal of Cleaner Production*, with 724 total citations, followed in third place by *Construction and Building Materials*, with 719 total citations.

**Figure 2:**
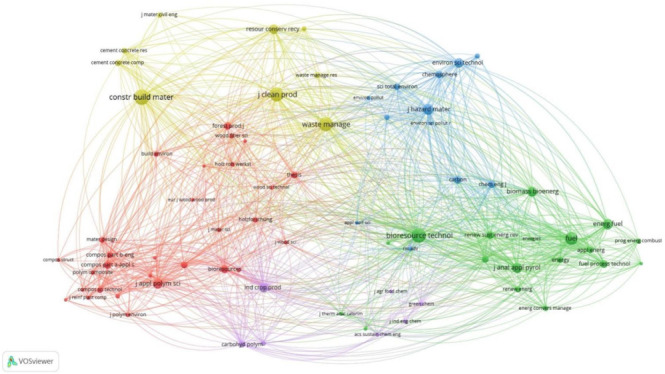
The co-citation network analysis of main sources of articles.

For a better understanding of the impact of the journals in the research field, a journal co-citation analysis map was generated using VOSviewer (Figure 2). The map is composed of a series of nodes and lines indicating the relative number of co-citations for a certain journal and their interconnections. The size of the bubble indicates the number of co-citations; the greater the size, the greater the number of co-citations for that journal. The position of the journals on the map is given by their co-citation frequency [12]. The grouping of the journals indicates a series of similarities of the published articles. The map also contains a series of lines that are used to indicate the links of co-citations between journals. The VOSviewer software uses colors for better visualization of the data, so for each group of journals, a certain color is being used. The journals sharing the same color are considered to have similarities in their published articles [15].

The generation of the map is based on a total of 5221 sources; the minimum number of citations of a source was set to 30, resulting in a number of 71 sources that met the threshold. The journal co-citation analysis generated four distinct and coherent clusters of journals on the network map. The cluster with the greatest number of items, 29 items, is the red cluster. In this cluster are journals such as *Forest Products Journal*, *Wood and Fiber Science*, the *Journal of Applied Polymer Science*, *Composites Part A: Applied Science and Manufacturing* and *Composites Part B: Engineering.* The journal with the greatest number of direct citations 192 is the *Journal of Applied Polymers Science*, which has a total link strength of 3948.

The second cluster by number of items is the green one, with 18 items. In this cluster, we can find journals such as *Bioresource Technology*, *Fuel*, *Energy Fuel*, *Energy*, *Renewable and Sustainable Energy Reviews* and the *Journal of Analytical and Applied Pyrolysis*. In this cluster, four journals stand out. The journal with the greatest number of direct citations is *Bioresource Technology*, having 387 direct citations and a total link strength of 9138. The second journal of importance in this cluster is *Fuel*, with 316 direct citations and a total link strength of 6823. Another two journals having around 200 direct citations are the *Journal* *of Analytical and Applied Pyrolysis*, with 200 direct citations and a total link strength of 4874, and the journal *Energy Fuels*, with 199 direct citations and a total link strength of 4292.

The third cluster by number of items, with 12 items, is the blue cluster. In this cluster, we find journals such as the *Journal of Hazardous Materials*, *Chemosphere* and *Environmental Science & Technology*. The most influential journal from this cluster is the *Journal of Hazardous Materials*, with 222 direct citations and a total link strength of 4864.

The fourth cluster, with nine items, is the yellow one. The journals grouped here are *Waste Management*, the *Journal of Cleaner Production*, *Construction and Building Materials* and *Resources, Conservation & Recycling*. Even if the number of journals grouped in this cluster is very small, this cluster has three of the most highlighted journals on the entire map. The journal with the highest number of direct citations (430) is the journal *Construction and Building Materials*, with a total link strength of 7093. The journal situated close to the center of the map, with 373 direct citations and a total link strength of 7376, is the journal *Waste Management*. The third journal highlighted on the map is the *Journal of Cleaner Production*, with 361 direct citations and a total link strength of 7790.

The last cluster, having only five items, is the purple one. The journals grouped here are *Industrial Crops and Products*, with 170 citations and a total link strength of 4469; *Carbohydrate Polymers*; *Cellulose* and *Green Chemistry*, all of them having few citations.

From the sources of information point of view, the co-citation analysis revealed five main directions of research according to the journals where the articles selected for the study were published. The first category is represented by the study of wood from its composition, at the cellular level; this is the group of journals grouped in the red cluster in Figure 2. Another direction of research is represented by the use of wood as a fuel solution, in particular to generate energy, and is the green category of journals. The third category is represented by journals that publish papers dealing with the use of wood as a building material, either in the form of construction elements or as building materials made out of wood waste.

Another category of journals approaches the subject of wood waste from the ecological point of view, dealing with the environmental issues related to the use of wood. The last category, a small category, focuses mainly on the chemical part of wood, from agricultural food chemistry to green chemistry solutions.

### 3.3. The Topical Focus in the Wood Waste Field of Research

**Figure 3:**
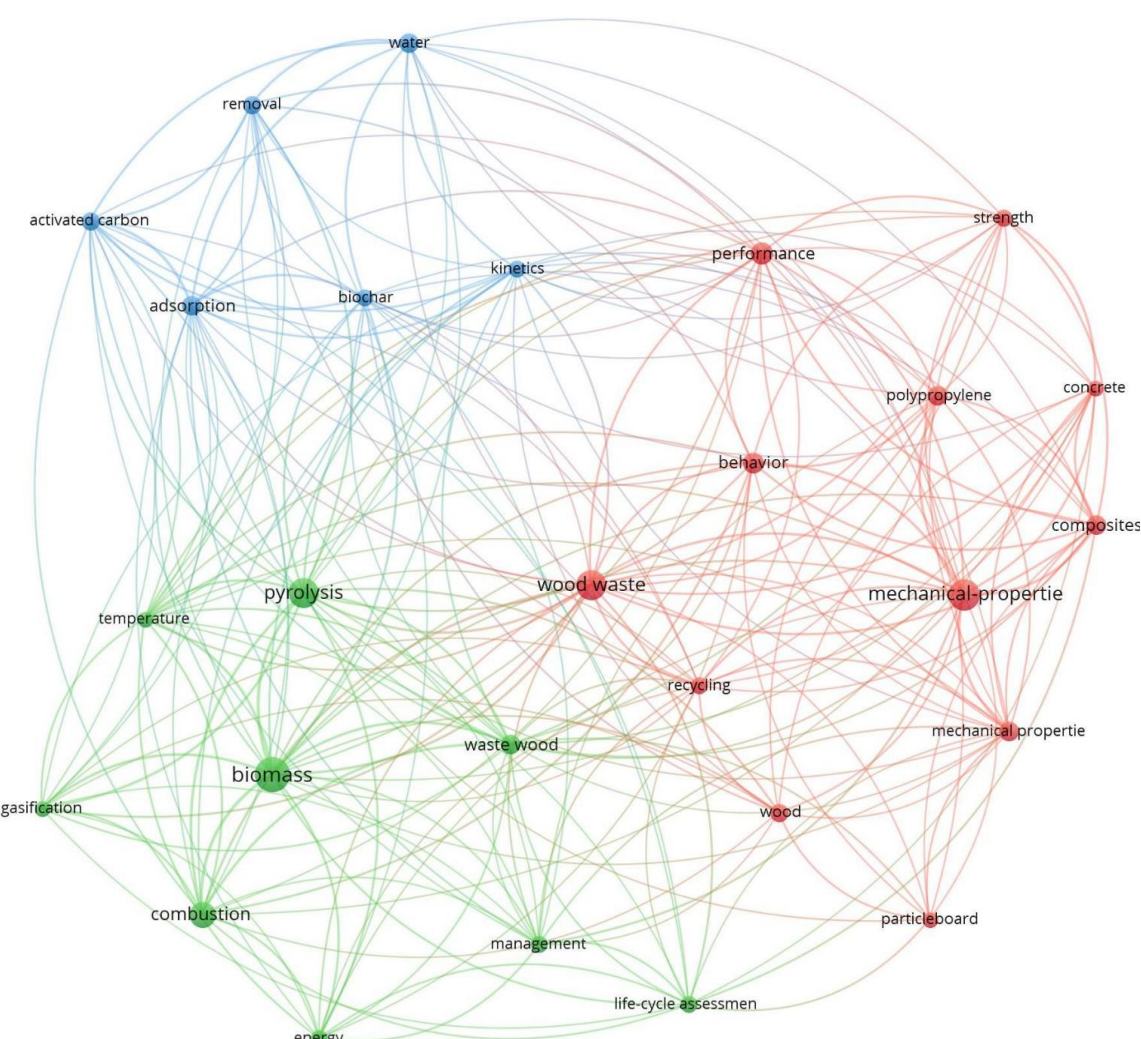
The keywords co-occurrence network map.

In the next phase of the research, a keyword co-occurrence analysis was performed (Figure 3). As in the case of journal analysis, the output of the keyword co-occurrence analysis is a network map highlighting the most used keywords, grouping the closely related words and showing their relations with the other keywords. In their work, researchers Zupic and Cater [12] explain that we can interpret the concepts described by the keywords as being closely related when there is a certain co-occurrence of the words in documents. The results of the keyword co-occurrence analysis indicate the most used keywords by the authors and can also indicate trends and patterns in the studied area [16,17].

The keywords co-occurrence map was generated based on 2240 keywords found in the articles selected for this study. By establishing a limit of 15 minimum occurrences for the keyword to be included in the results, just 27 keywords met the threshold. The keyword co-occurrence analysis also offers important insight regarding other rising topics related to the studied area [18]. The main aspects visualized in the keyword co-occurrence map are the occurrence of the keywords based on their prevalence and the evolution in time of their popularity [19,20].

On the network map can be observed three clusters for the keywords occurrence analysis. According to the sizes of the clusters, the red cluster has twelve items, the green cluster has nine items and the blue cluster has six items.

The center of the map, as expected, is dominated by the keyword “wood waste”, from the red cluster, with an occurrence of 45 times and a total link strength of 74. Other keywords highlighted on the map are “biomass”, “combustion”, “pyrolysis” and “waste wood” from the green cluster; “kinetics”, “adsorption”, “biochar” and “activated carbon” from the blue cluster; and “behavior”, “performance”, “strength” and “mechanical properties” from the red cluster.

The keywords analysis revealed an evolution in the main approaches regarding the use of wood. In the first phase of the research, the keywords were grouped into three main categories, which confirms the grouping from the journal analysis. One category of keywords grouped words such as wood waste, recycling, mechanical properties, concrete, strength, wood and performance. All of these words are used in the construction industry and are specific to building materials characteristics, so these can be considered the group of papers oriented towards the use of wood as a building material. Another category is formed by keywords such as biomass, combustion, gasification, pyrolysis, temperature and waste wood. In this category, we can find papers which approach the wood study from the perspective of wood generating energy. The last category revealed by the keywords analysis is a category of research oriented towards the study of wood as a living plant and its ecological potential. Here, we find words such as activated carbon, adsorption, biochar, kinetics and water.

At this point, it is important to clarify some aspects regarding the presence of two keywords, “wood waste” and “waste wood”, in different clusters on the network map. Even if these initially seem like a mistake because both of them are referring to waste produced by the use of wood at different stages, a distinction must be made. The term “wood waste” is usually used when it expresses a quantity of scraps or some pieces of wood that cannot be used in construction for different reasons, either due to dimensional limitations or because they are leftovers from a construction element or from different temporary structures, e.g., formworks and so on. The same words are used to express the quantity of wood resulting from pallets, wood used in the transport industry or other waste resulting from the use of wood in various forms. When we speak about pieces of wood of low quality (which cannot be used for something else), contaminated wood or pieces of wood from low-quality species (which are mainly used as fuel for fire), in these cases, we use the term “waste wood”; that is why the keyword “wood waste” is grouped in the building materials category and the keyword “waste wood” is placed in the category of energy producer.

#### 3.3.1. Trend Topic Analysis

**Figure 4:**
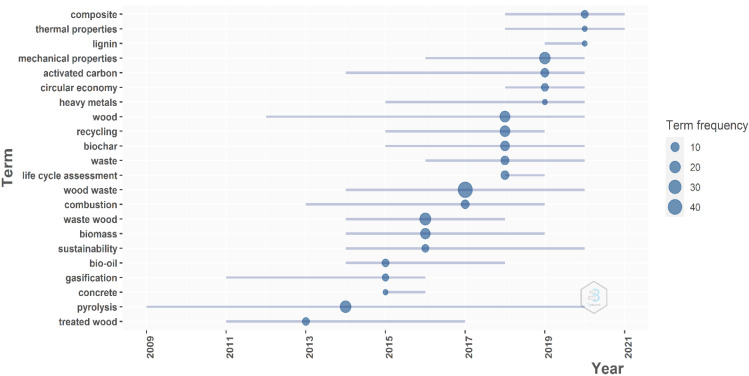
The trend topics plot.

Analyzing further the keywords used by authors to better express their topic interests, a trend topic analysis was made. The analysis was made using Bibliometrix software, and the graph from Figure 4 was generated. The trend topic plot, constructed based on the main keywords used by the authors of the papers, reveals an evolution and a change in the approach related to the use of wood in the period from 2008 to 2021.

In the generation of the trend topics, only papers published from 2008 to 2021 were introduced. Other graphical parameters refer to the use of the author’s keywords field, with a minimum word frequency of five, and the number of words to be considered per year was set to five. In these conditions, from Figure 4, the main keywords used in each year can be observed. The lines represent the years when that word was used; highlighted by a bubble on the line is the most frequent time that that word was used and in what year. The size of the bubbles represents the frequency of use for each term; the bigger the bubble, the higher the frequency of use. The results are similar to the results shown in the VOSviewer. The most frequently used term is “wood waste”, with a maximum frequency of 45 in 2017.

It is also interesting to observe how the trend was generated. If in 2013, the most frequent word was “treated wood”, then the research evolved, and in 2014, “pyrolysis” was explored more, followed by words such as “bio-oil”, “biomass”, “sustainability” and “wood waste”. In the last several years, words such as “recycling”, “circular economy”, “mechanical proprieties” and “composite” appear. Just by reading these keywords, it can be seen that although wood waste was mainly analyzed in the early 2010s from the combustible point of view as a method to generate energy, in the last five years, in the context of rising to the challenge of environmental protection, the researchers have tried to look at wood waste as a solution to be used in the construction industry as building materials.

The trend topic shows a change point in the approaches of the wood topics. In the first part of the time interval, the most used keywords indicate that most topics were related to the use of wood as an energy producer. Until 2017, the most used keywords were “treated wood”, “pyrolysis”, “gasification”, “biomass” and “combustion”. After 2017, other words appear to be more used, such as “biochar”, “recycling”, “circular economy”, “mechanical proprieties”, “thermal proprieties” and “composite”. All of these indicate the switch in the last five years from the consideration of wood as just a source to generate heat or energy to the use of wood as a building material and thus as a solution to decrease the environmental impact of buildings and of the construction industry in general.

#### 3.3.2. Thematic Evolution of the Keywords

**Figure 5:**
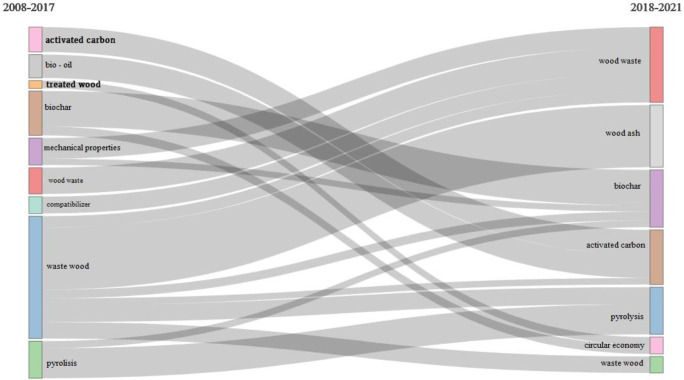
The thematic evolution of the keywords.

Having these results and observing a turning point in the evolution of the trend topic, in the next phase, also with the use of Bibliometrix software, a thematic evolution of the keywords was generated (Figure 5).

The map was constructed considering the turning point of the year 2017; thus, two time intervals were generated: 2008 to 2017 and 2018 to 2021. The thematic evolution of the keywords was generated with the following parameters: the number of words was set to 250; minimum cluster frequency to 5; weight index inclusion as index weighted by word-occurrence; minimum weight index to 0.1 and number of labels for each cluster to 1.

The results shown in Figure 5 confirm the results from Figure 4. If, in the period of 2008–2017, the most used keywords were “activated carbon”, “bio-oil” and “treated wood”, in the following time period of 2018 to 2021, the importance of activated carbon and bio-oil decreased. The “treated wood” disappears, and it is replaced by “circular economy”, which also includes a part of “biochar”. In the last several years, we can observe the increased importance given to wood waste products, especially in the form of building materials to be used in future constructions to decrease the carbon footprint of buildings. Another important category of materials was oriented towards topics related to waste wood, and it can be seen that the approaches are focused on solutions to generate energy either as pyrolysis or as biochar, but a large part of the approaches are also oriented towards the use of wood ash, continuing the cycle. Most of the use of ash is also in the building materials segment; ash is used as a component of concrete mix in order to decrease the energy demands in producing it. From this analysis, an increasing importance given to topics related to the production of new building materials made out of wood waste can be observed, as well as to finding ways to use waste wood other than as a heat generator.

#### 3.3.3. Topic Dendrogram

**Figure 6:**
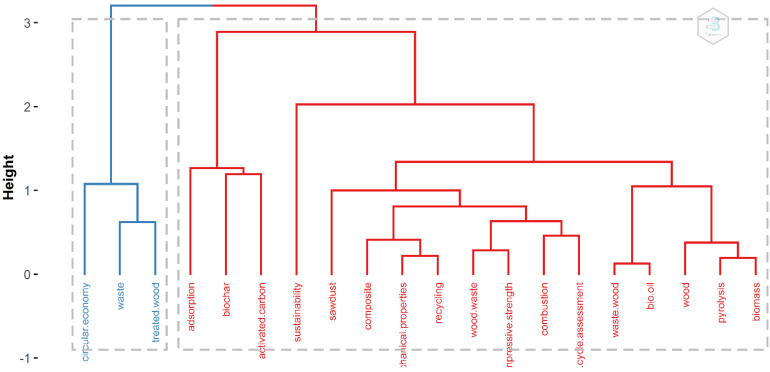
The topic dendrogram.

The conceptual structure of the used keywords, and thus the essence of the main topics studied in the field of wood waste, can go further on, and in Figure 6, the topic dendrogram is presented.

The topic dendrogram was generated based on factorial analysis with the following parameters: the method to generate the dendrogram was multiple correspondence analysis; field author keywords; the number of terms was set at 20 and the number of clusters was allowed to be chosen automatically by the program. The topics were grouped into two clusters. The first cluster is formed by “circular economy”, “waste” and “treated wood”, and in the second cluster, “adsorption” was coupled with “biochar” and “activated carbon”. The next part is formed by “sustainability” and all other keywords that contribute to the sustainability challenges.

In general, it can be observed that the main ideas from the previous analysis were confirmed; thus, in the first group are issues related to the implementation of the circular economy by using waste and treated wood products. In the other group can be found topics related to the properties of wood to store carbon and to obtain biochar, as well as the sustainability aspects from the two points of view, wood as a building material and wood as a combustion material.

### 3.4. Research Topics Addressed in the Wood Waste Field of Research

As it can be seen from the above sections, the topics addressed in the wood waste field of research are focused on several directions. Almost all the research is based on the situation created by the rapid growth of population and the need to provide better waste management, to create more energy to maintain a constant temperature, especially in the cold periods of the year, and to offer more living spaces. Disposal of wood waste in landfills also creates other problems due to the fact that wood disposal could result in methane emissions and/or leaching of hazardous constituents polluting water or soil. At the same time, large quantities of unused wood waste are produced in both the extraction of raw material and its transformation into building materials, as well as in construction technology. This means that it is a low-cost source of woody biomass [21] that makes it suitable to be used in various forms to decrease environmental impacts and also to decrease the cost of elements.

The focus of this study was on wood waste, so studies such as [22,23], which deal with the same situations [24,25] but are focusing on food waste and sludge or rice straws, were not fully analyzed. Other studies, such as [26], take into consideration forest residues and agricultural wastes with the purpose of creating biomass. The transformation of agro industrial waste into building insulation material [27] is also analyzed.

Considering that the unprecedented growth level of the modern society burdens the anthroposphere with serious resource supply risks and waste generation, the idea of a “circular economy” is starting to be more and more feasible, and the implementation of the three Rs (reduce, reuse, recycle) is beginning to take shape [7,28]. In the construction industry, more and more studies [28,29] are adhering to this challenge and focusing on the development of various solutions to better use construction [30] and demolition waste [9,31]. The study in [32] explores this idea and analyzes the wood waste hierarchy framework in the European region.

From a building materials point of view, a big category of research is dedicated to the use of wood waste in particleboard production. In [33], it is stated that “the production of high-quality boards from alternative lignocellulosic raw materials is feasible and brings a series of ecological and economic benefits”. Particleboard can be obtained by using waste wood formwork, as shown in [34,35], and can be transformed into cement-bonded particleboard using MOC (magnesium oxide cement) as a green cementitious binder; thus, it presents a practical and eco-friendly management option for construction wood waste. In the particleboard mix, other materials besides the wood waste can also be added, e.g., tire fibers or biomass ash [36].

Another use of wood waste is in the mixture of building materials, such as concrete or mortar. The study in [37] addresses the use of waste aggregate in gypsum mortars. It is shown that even if the use of waste decreases the mechanical strength of the product, in some situations it can be used properly. Other ways of using wood waste is in the form of ash, which can be added to a mortar [38,39] or concrete mixture [40,41]. The researchers in [42] state that “wood waste ash seems to be promising to use as pozzolanic partial replacement material for cement, with no strength loss and leading to enhanced durability and thus contributing to sustainable construction”. In [43], it is demonstrated that “the utilization of waste wood materials both in the form of wood powders and wood fibers provides a greener alternative for the waste recycling of industrial debris with respect to the existing waste management options, as well as saves natural resources and the CO_2_ emissions required to produce the raw materials for the manufacture of cement mortars”. The use of biochar as a component in the concrete mixture is studied [44], and it is shown that, if used in an optimal percentage, the addition of biochar can have positive effect on the concrete and thus can help to develop the circular economy building material.

The use of wood waste in the composition of low-strength concrete materials is addressed also [45,46]. In [46], it is shown that “proper utilization of sawdust in concrete will conserve the environment by reducing the use of natural resources, reducing the volume of waste material, and reducing CO_2_ emissions”. The new material Wood-Crete, developed by the researchers of [45], indicates the possibility of using wood waste and concrete products for in-fills for wall panels and hollow blocks or for thermal insulating material. The good properties of the wood wool–cement composite material are highlighted by [47], which shows that “the material presents excellent mechanical, chemical and biological properties. However, the understanding of its mechanical behavior is rather limited”. The researcher developed a hierarchically structured model to describe the mechanical behavior under compressive stress. The wood waste from pallets was also studied as a replacement for spruce in wood wool–cement composite. The researchers [48] indicate that the use of waste wood is successful up to 50%, but above these limits, “the strand morphology is too heterogeneous to guarantee a good reinforcement”.

The incompatibility between wood and cement is analyzed by [49], and it is proven that “the alkaline hydrolysis was found as the most effective treatment for the suppression of inhibitory substances and the highest decrease on mechanical properties of resulting composites”. There is obviously a need to find a proper balance in the wood-and-cement composition, which is quite difficult to determine. A solution to this problem is proposed by [50] by developing a model to calculate the competitive water uptake of natural strands (wood wool) and cement in wood–cement composites.

The same conclusion of a proper and optimum ratio between cement and wood was observed in [51], where the researchers studied the possibility of using demolition waste in unfired bricks manufacturing. They underline that “the substitution of a natural soil by recycled targets modifies the target binder chemical reactions. This could be taken into account for the optimization of the target formulation and binder kind and dosage selection to optimize the unfired brick manufacturing from technical and environmental points of view”.

The thermal insulation properties of the wood products [52,53] are another topic addressed in the literature [54,55]. In [56], it is shown that the use of 5% date palm fibers in gypsum mix can contribute to the obtention of a composite material with good mechanical and thermal proprieties. The possibility of using low-density materials for thermal insulation is presented in [57]. The use of various wastes in cementitious composite is studied by [58], which highlights the use of these products in the case of construction elements with low strength requirements and the necessity to use local waste in order to limit the quantity of energy used by transporting.

The use of wood waste in the form of biochar is another large category of research [59]. The possibility of using wood waste as a renewable energy resource is another topic approached in the literature. In [60,61], the importance of better using wood biomass as a renewable source of energy, as well as the implication of developing modern ways of production and utilization of woody biomass, is studied. The combustion behavior of wood waste was studied in [62], and they underline that “waste wood seem to be suitable with combustion. However, the high nitrogen content gives rise to higher NOx production and would require more severe gas cleaning. Moreover, the presence of external pollution may raise specific issues due to the presence of fine particles, which make combustion more difficult, and may impose a separation step between fine and coarse fractions”.

Waste wood materials come from different sources, and one of the biggest problems is generated by the reuse of contaminated wood materials [63]. One approach is to use industrial wood waste, for example wood-based panels, in pyrolysis technology and transform them into pyrolytic gas, liquid bio-oil and solid activated carbon [64]. Other studies shows that the use of glued wood waste as a combustion material is very problematic but can be done in medium or large plants ”where with economic investments it is possible to design cutting-edge combustors” [65].

The use of wood waste as adsorbents from biomass waste is also studied. Wood-derived activated carbons are efficient adsorbents that can separate a wide range of organic and inorganic pollutants. In [66], it is shown that “conversion of abundant wood biomass into activated carbon can have several applications such as manufacturing of gas mask filter, drinking water filter bed, municipal wastewater treatment plant, treatment of dyes and metal-ions containing industrial effluent”.

One research direction is focusing on the use of wood composite material **polymers** [67]. The studies show good behavior and a multitude of uses for wood polymer products, but there is still a need to develop a more cost-effective fabrication process for the mass production of wood composites [67].

## 4. Conclusions

The purpose of this paper was to investigate the field of wood waste and identify if, in the context of more and more awareness of the impact of the human activity on the environment and in the global attempt to implement the 17 Sustainable Development Goals proposed by the 2030 Sustainable Development Agenda, the use of wood waste as a building material can be a solution to achieve the sustainable development goals.

The major conclusions resulted from this study can be summarized as follows:

- Wood is the only renewable and ecological material that can not only reduce the production of CO_2_ but also store large parts of it. In order to benefit from the use of wood, we need to consider two main aspects. The first one is to always use wood from sustainably managed forests, and the second one is to find methods to use as much as possible from the tree, mainly to decrease the amount of wood waste.
- There has been an increased interest in the topic of using wood waste in the last several years, especially after 2018, and a switch from the consideration of wood waste as just a source to generate heat and energy to the use of wood waste as a building material, and thus a solution to decrease the environmental impact of buildings and of the construction industry in general, can also be observed.
- All over the world, large quantities of wood waste are produced, and in most cases, these quantities are not used. Their disposal in landfills creates bigger ecological problems due to the decomposition of wood and the generation of compounds that pollute the air, soil or water. As such, besides resolving a pollution situation, we are dealing with a very low-cost raw material that can be used in various ways.
- As a building material, one method of using wood waste is the production of particleboards. The main limitation of this use of the waste is that a certain degree of strength is necessary for the waste, and it must be an uncontaminated wood that can be shredded into various sizes and then used in the particleboard mix. However, the use of wood waste for particleboard production is a viable solution, and it has good results.
- A second method of using wood waste as a building material is the use of waste in concrete or mortar mixtures. Here we have two main approaches: one is using the raw material in shape of small particles, e.g., sawdust, and the other is using the remains from burning the wood (the ash) in the concrete composition. The main limitation in this approach is the incompatibility between the cement and the wood. The cement needs a certain humidity to activate and to increase its strength, while the wood is acting as an absorbance of humidity, and thus the concrete obtained from this combination can have significant decrease in mechanical strength; additionally, a large number of cracks appear on the surface of the elements. Many studies show that there is the need for a proper mix of ingredients and find that ratios still need further investigation.
- The general use of building materials made of wood and cement mixtures is as nonstructural elements or filling elements. Another use of this mixture with good results is in the thermal insulation of buildings.
- A solution to the wood–cement incompatibility can be the replacement of portland cement with a new type of cement, magnesium oxychloride cement (MOC), which has good mechanical proprieties and does not depend on the presence of water. This is a direction that can be explored more in the future.

The general conclusion drawn from this study is that the use of wood waste is becoming a more and more interesting topic of research and has many directions that can be developed, from the use of wood waste to generate renewable energy to the development of new sustainable building materials. The forest, and implicitly the wood, is an immense natural treasure, and finding ways to better use it by developing methods for the integral use of the wood from trees, including the remains, can surely lead not only to the fulfillment of the Sustainable Development Goals but also to the better welfare of all humankind.

## Figures and Tables

**Table 1:** Search criteria.

Search	Search Criteria	Nb. of Articles
Title	“wood waste”	1348
Document type	Article, Review	1059
Language	English	956
Research area	Material science, Engineering, Construction Building Technology	542

**Table 2:** Top 10 journals by the number of published articles with wood waste topic.

Nr.crt	Journal Name	Article No.	TC
1	*Journal of Cleaner Production*	31	724
2	*Waste Management*	29	899
3	*Bioresources*	22	51
4	*Construction and Building Materials*	20	719
5	*Journal of Hazardous Materials*	14	481
6	*Fuel*	13	401
7	*Energy & Fuels*	11	185
8	*Polymer Composites*	9	216
9	*Resources, Conservation & Recycling*	9	271
10	*Forest Products Journal*	8	70

## Data Availability

No new data were created or analyzed in this study. Data sharing is not applicable to this article.
